# A2PM-VINS: A Visual–Inertial SLAM Method Based on Area-to-Point Matching

**DOI:** 10.3390/s26103071

**Published:** 2026-05-13

**Authors:** Mengxing Ma, Zengao Jiang, Yunhai Yan, Jianing Tang, Yunhao Chen

**Affiliations:** 1 School of Electrical and Information Engineering, Yunnan Minzu University, Kunming 650500, China; mamengxing@ymu.edu.cn (M.M.); jpppppp@ymu.edu.cn (Z.J.); 041749@ymu.edu.cn (J.T.); 041967@ynni.edu.cn (Y.C.); 2 Yunnan Key Laboratory of Unmanned Autonomous Systems, Kunming 650500, China

**Keywords:** visual–inertial SLAM, repetitive textures, area-to-point hierarchical matching, feature selection, sliding-window optimization

## Abstract

**Highlights:**

**What are the main findings?**

- Introducing Area-to-Point hierarchical matching into the front end can suppress cross-region mismatches in repetitive-texture scenes and improve the reliability of front-end observations.
- The Anchor–Explorer feature selection strategy can identify high-quality features that balance stability and geometric contribution, thereby enhancing the effectiveness of the observations used in back-end optimization.

**What are the implications of the main findings?**

- In complex degraded environments with repetitive textures, low illumination, and weak textures, improving front-end observation quality and track continuity is more beneficial to visual–inertial state estimation than simply increasing the number of candidate features.
- The proposed method provides an extensible front-end/back-end collaborative design for visual–inertial SLAM in complex degraded environments.

**Abstract:**

The localization performance of visual–inertial simultaneous localization and mapping (VI-SLAM) strongly depends on front-end feature matching. In degraded scenes with low illumination, repetitive textures, and weak textures, traditional geometric front ends often suffer from sparse features and mismatches, resulting in unstable state estimation. To address this issue, this paper proposes Area-to-Point Matching Visual–Inertial SLAM (A2PM-VINS), a visual–inertial SLAM method based on Area-to-Point matching. The method introduces Area-to-Point hierarchical matching and a kinematic temporal inheritance mechanism to improve matching reliability and track continuity, and further designs an Anchor–Explorer feature selection strategy to retain features with higher geometric value for back-end optimization. In addition, a Sub-Window Consistency (SWC) weighting strategy is incorporated into the back end to suppress geometrically deceptive observations with poor temporal continuity and geometric consistency. Experiments on the European Robotics Challenge Micro Aerial Vehicle (EuRoC MAV) dataset show that A2PM-VINS achieves superior or competitive localization accuracy on multiple challenging sequences. The absolute trajectory errors on MH_04 and MH_05 are 0.0983 m and 0.1191 m, respectively, and stable tracking is maintained on V2_02, where VINS-Fusion fails. These results show that the proposed method effectively improves the robustness of visual–inertial state estimation in complex degraded environments.

## 1. Introduction

Visual–inertial simultaneous localization and mapping (VI-SLAM) is one of the key technologies for pose estimation, environment perception, and global consistency maintenance in mobile platforms. In environments where global navigation signals are limited or unavailable, visual–inertial systems (VINS) based on cameras and inertial measurement units (IMUs) have been widely applied in mobile robots, unmanned aerial vehicles, autonomous inspection, and indoor navigation because of their low hardware cost, strong sensor complementarity, and suitability for compact deployment. After years of development, visual–inertial SLAM has developed into a relatively mature technical framework, mainly including two research routes: filtering-based recursive estimation and nonlinear optimization-based batch estimation. In the filtering route, MSCKF and OpenVINS respectively represent visual–inertial estimation methods and open-source research platforms based on the multi-state constraint extended Kalman filter [1,2]. In the optimization route, OKVIS [3], VINS-Mono [4], VINS-Fusion [5], and ORB-SLAM3 [6] have achieved remarkable progress in localization accuracy and system performance by jointly exploiting visual observations and inertial constraints. In addition, SVO has verified the effectiveness of semi-direct methods in visual odometry [7]. Although these methods perform well in texture-rich and well-lit environments, their front ends still generally rely on low-level geometric observations such as corner points, local descriptors, or optical-flow tracking. Therefore, they remain prone to observation degradation and matching failure in low-light, weak-texture, and repetitive-texture scenes.

In practical industrial and inspection scenarios, visual degradation is widespread. On the one hand, low illumination and weak textures significantly reduce image contrast, leading to a decrease in the number of usable features and an increase in observation noise. On the other hand, repetitive textures formed by pipes, grilles, window frames, and regularly arranged structures reduce the distinctiveness of local appearance features and increase the risk of incorrect matching. When the system simultaneously encounters repetitive textures, weak textures, and rapid viewpoint changes, front-end feature matching can easily fail, which further causes the back-end optimization to be disturbed by erroneous observations, resulting in local trajectory deviation, increased accumulated drift, or even tracking failure. Therefore, how to improve the robustness and state-estimation stability of visual–inertial systems in complex degraded environments remains a key issue in current research.

In recent years, advances in deep learning for feature extraction and matching have provided new opportunities to overcome the limitations of traditional geometric methods in degraded environments. In learning-based feature extraction, SuperPoint [8], as a representative self-supervised keypoint and descriptor network, has laid the foundation for learning-based front ends. In learning-based matching, Local Feature Transformer (LoFTR) improves correspondence robustness in weak-texture regions through detector-free matching and global context modeling [9], while LightGlue further improves matching efficiency by using an adaptive feature filtering mechanism [10]. In addition, dense geometric matching methods such as DKM have also promoted robust correspondence research under large viewpoint changes and low-texture conditions [11]. To address the highly challenging problem of repetitive textures in industrial scenes, Area-to-Point Matching (A2PM) first alleviates local ambiguity in point matching through Area-to-Point hierarchical constraints [12]. On this basis, the same author team further proposed Matching Everything by Segmenting Anything (MESA) to strengthen region-level correspondence modeling [13]. At the system-integration level, VINS-FEN attempts to embed a convolutional neural network feature extraction module into the VINS front end to improve stability under illumination changes [14]; SuperVINS integrates SuperPoint and LightGlue into the VINS-Fusion framework and shows good adaptability in low-light scenarios [15]; D-VINS mainly introduces deep features into loop closure detection to enhance relocalization capability [16].

In addition to point features and dense matching, recent studies have also used line features, point–line structural features, and hybrid matching strategies to improve the stability of visual–inertial systems in degraded environments. PL-VINS introduces joint point–line constraints into VINS-Mono to use structural line segments as a supplement to point features in weak-texture regions [17]. DeepLine-VIO further uses learning-based line features to improve visual–inertial estimation robustness under low-texture and illumination-changing conditions [18]. XR-VIO improves front-end tracking stability from two aspects: visual–inertial initialization and hybrid optical-flow/descriptor matching. AirSLAM [19,20], a recent illumination-robust visual SLAM system, combines learning-based point–line feature extraction with conventional back-end optimization to handle short-term and long-term illumination changes. AirSLAM shows the value of combining learning-based point–line features, geometric optimization, and efficient deployment for improving localization stability under complex illumination conditions [20].

These studies improve the robustness of visual or visual–inertial systems in degraded environments from different perspectives, and they also show that front-end observation quality, structural feature utilization, and matching stability are important for state estimation. Compared with these works, the focus of this paper is different. Point–line fusion and learning-based line-feature methods mainly introduce structural cues to enhance visual observations, while hybrid matching methods focus more on improving continuous tracking and initialization stability. This paper instead focuses on how region-level correspondences can organize point-level matching in repetitive-texture and weak-texture scenes, thereby reducing cross-region incorrect associations. At the same time, deep front ends usually generate many candidate features. Without effective filtering for back-end optimization, information redundancy increases computational load and may affect the stability of sliding-window optimization. Moreover, A2PM is mainly designed for image-pair matching [12]. Although it can alleviate local ambiguity caused by repetitive textures at the spatial level, its original framework does not specifically address track inheritance in continuous video streams. Therefore, this paper further designs region-constrained matching, track inheritance across consecutive frame pairs, and candidate feature selection to improve the stability of visual–inertial state estimation in complex degraded environments.

This paper argues that, in complex degraded environments involving repetitive textures, low illumination, and weak textures, improving front-end matching reliability, trajectory continuity, and feature quality is more beneficial to visual–inertial state estimation than simply increasing the number of candidate features. Based on this understanding, this paper proposes A2PM-VINS, a visual–inertial SLAM method based on Area-to-Point matching. Built upon VINS-Fusion [5], the proposed method introduces the Area-to-Point hierarchical matching framework of A2PM into the front end and further incorporates a kinematic temporal inheritance mechanism to enhance feature-track continuity in video streams [12]. In addition, an Anchor–Explorer feature selection mechanism is designed to select high-quality features with greater geometric contribution to back-end optimization from a large number of candidate features, thereby improving feature distribution and optimization conditions. Furthermore, a Sub-Window Consistency (SWC) weighting strategy is introduced into the back end to suppress geometrically deceptive observations with poor continuity and geometric consistency within local temporal windows. Through the above joint front-end and back-end design, this paper aims to improve state-estimation performance in complex degraded environments from three aspects, namely front-end matching quality, feature-selection strategy, and robust back-end optimization. Experimental results on the EuRoC dataset further show that the proposed method achieves superior or competitive trajectory accuracy on multiple challenging sequences.

The main contributions of this paper are summarized as follows. (1) To address region ambiguity in point-level matching under repetitive textures, this paper introduces the area-to-point hierarchical matching framework of A2PM in the front end and further combines it with a temporal inheritance mechanism based on historical track priors. Under region-level prior constraints, this mechanism performs point-level matching and maintains internal track IDs across consecutive frame pairs through a shared-frame association strategy, thereby reducing incorrect matching and track fragmentation in repetitive-texture scenes. (2) To address the problem of numerous and redundant candidate features, this paper proposes an Anchor–Explorer feature selection mechanism. This mechanism builds a compact high-quality feature subset according to the geometric contribution and constraint value of features, while controlling the total number of features, improving feature distribution, and improving back-end optimization stability. (3) To suppress geometrically deceptive observations that may enter the sliding window and affect state estimation in local degraded segments, this paper proposes a back-end weighting strategy based on sub-window consistency. This strategy retrospectively evaluates the temporal continuity and geometric consistency of landmark observations in local time windows and downweights observations with poor geometric consistency, thereby further improving state-estimation stability in complex degraded scenes.

## 2. Materials and Methods

### 2.1. System Overview

**Figure 1:**
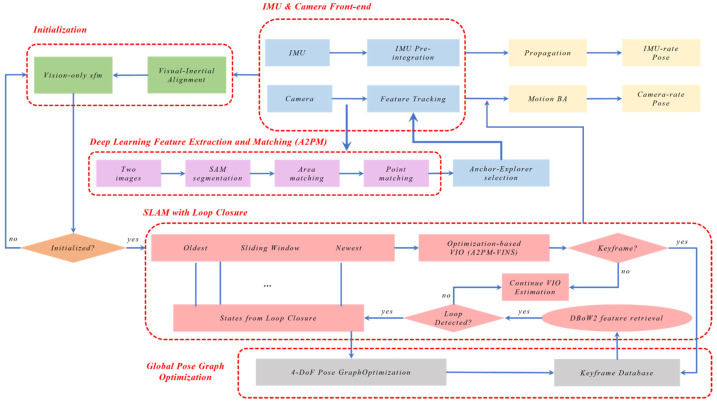
Overall architecture of Area-to-Point Matching Visual–Inertial SLAM (A2PM-VINS). The framework consists of a front-end A2PM Area-to-Point hierarchical matching and dual-role feature selection module, a back-end sliding-window optimization module, and a loop closure detection and global pose graph optimization module. The arrows indicate the data flow between modules, and the different colors are used to distinguish different processing modules or stages.

A2PM-VINS is built upon VINS-Fusion for visual–inertial SLAM tasks in degraded scenes and repetitive-texture environments. As shown in Figure 1, the overall system mainly consists of a front-end A2PM Area-to-Point hierarchical matching and feature selection module, a back-end sliding-window state estimation module, and a loop closure detection and global pose graph optimization module. The goal of this system is to improve front-end matching reliability, feature-track continuity, and the robustness of back-end state estimation in complex degraded environments while keeping the basic back-end solver framework of VINS-Fusion unchanged.

Specifically, this paper improves the system from two aspects, namely front-end matching and back-end optimization. First, the A2PM Area-to-Point hierarchical matching framework is introduced into the front end, where region-level correspondences are used to constrain the search range of point-wise matching so as to reduce cross-region mismatches in repetitive-texture environments. Second, to address the difficulty of directly applying the original A2PM framework to continuous image sequences, a temporal inheritance mechanism is further designed so that frame-to-frame matching results can stably continue historical tracks. Third, considering that deep front ends generate a large number of candidate features with uneven quality, an Anchor–Explorer (A–E) dual-role feature selection mechanism is proposed to preserve high-quality features that balance stability and geometric contribution under a fixed feature budget. Finally, a dynamic weighting strategy based on Sub-Window Consistency (SWC) is introduced into the back end, where the weights of visual observations are adaptively adjusted according to the temporal continuity and geometric consistency of tracks within a local temporal window, thereby suppressing geometrically deceptive observations in complex degraded scenes.

In implementation, the selected front-end features and the IMU preintegration constraints are jointly fed into the sliding-window optimization module of VINS-Fusion, while the loop closure detection and global pose graph optimization module is used to further suppress accumulated drift during long-term operation. Through the above design, A2PM-VINS organically combines region-constrained matching, a temporal inheritance mechanism, feature-quality-aware selection, and back-end consistency weighting without changing the overall solver framework of VINS-Fusion, thereby forming the visual–inertial SLAM system proposed in this paper.

### 2.2. A2PM with Trajectory Inheritance

To address the repetitive-texture problem commonly encountered in industrial scenes, this paper introduces A2PM into the front end as the basic matching framework [12]. A2PM adopts a coarse-to-fine two-stage matching strategy: region-level correspondences are first established, and point-wise matching is then performed within the paired regions. Region-level correspondences serve as geometric priors that constrain the search range of point-wise matching, thereby reducing region ambiguity caused by relying only on local appearance similarity.

In the implementation of this work, the input image is first segmented by Segment Anything Model (SAM) to generate the corresponding region masks [21]. Then, a region graph is constructed according to the spatial layout and adjacency relationships of the regions, and Densely Matching Everything by Segmenting Anything (DMESA) [22] is used to establish region-level correspondences. After the paired regions are obtained, the system no longer performs global point matching directly on the full image. Instead, local sub-images are cropped according to the boundaries of the matched regions so as to preserve more local details, and LoFTR is then applied within the corresponding region masks to complete point-wise matching [9]. This coarse-to-fine strategy can effectively suppress cross-region mismatches and is particularly suitable for scenes with large-scale repetitive textures, such as window frames, grilles, and box arrays. Because point-wise matching must satisfy the region-mask constraints, incorrect matches with similar appearance but lying across different regions can be directly removed in the front end.

**Figure 2:**
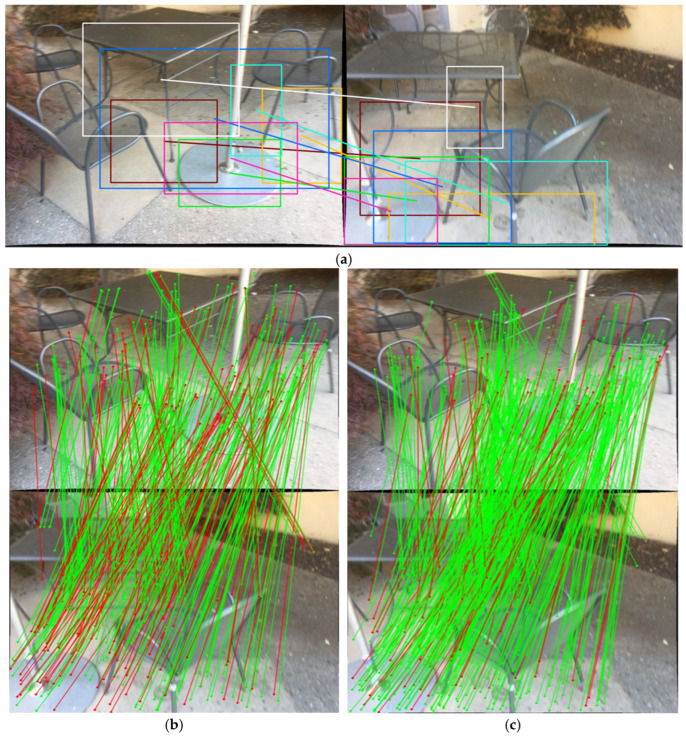
Illustration of A2PM area-to-point matching. (**a**) Inter-region matching result, where colored boxes indicate matched regions and colored lines indicate correspondences between paired regions; (**b**) point matching result; (**c**) point matching result under inter-region matching constraints.

To more clearly illustrate how region-level correspondences constrain the subsequent point-level matching process, Figure 2 shows the visualization result of A2PM area-to-point matching. Figure 2a shows the region-level correspondences obtained by SAM segmentation and DMESA region matching, where paired regions provide spatial priors for subsequent point-level matching. Figure 2b shows the original matching result obtained by directly applying the point matching network over the whole image. In repetitive-texture or appearance-similar regions, point-level matching without region priors is more likely to generate cross-region candidate associations. Figure 2c shows the point-level matching result under region-level correspondence constraints. In this case, LoFTR is only applied inside paired regions with established correspondences, so candidate correspondences are limited to local regions allowed by the region-level prior.

As shown in Figure 2, region-level correspondences in A2PM are not the final point matching results. Instead, they serve as spatial constraints before point-level matching. By first establishing region-level pairings and then applying LoFTR point-level matching inside paired regions, the system reduces incorrect candidate correspondences caused by repetitive appearance across the whole image. The retained matches are therefore more stable in terms of spatial distribution and geometric consistency. This process also provides more reliable front-end observations for the subsequent historical-track inheritance and back-end sliding-window optimization.

However, the original A2PM framework is mainly designed for image-pair matching and cannot directly handle the problem that feature tracks are difficult to stably continue in continuous image sequences. If adjacent-frame matching results are directly processed as independent image pairs, the same physical point may be repeatedly initialized in different frame pairs, leading to track fragmentation and weakening the use of continuous geometric constraints in the back end. Therefore, based on the adjacent-frame matching results of A2PM, this paper introduces a temporal inheritance mechanism based on historical track priors to maintain internal track IDs for candidate matches across consecutive frame pairs.

Specifically, after completing area-to-point matching for the previous adjacent image pair $\left(I_{k-1},I_{k}\right)$, the system stores the endpoint position of each established historical track in the shared frame $I_{k}$ and its internal track ID. For the *i*-th historical track, its endpoint position in the shared frame $I_{k}$ is denoted as $p_{k}^{i}$. During the matching association process for the current image pair $\left(I_{k},I_{k+1}\right)$, this endpoint position is used as the association reference position of the historical track in the shared frame $I_{k}$, namely:

(1) $$p^{\prime }=p_{k}^{i}$$

where $p^{\prime }$ denotes the historical track prior center in the current association step and is used to constrain the starting-point association of the current candidate match in the shared frame $I_{k}$. This historical track prior comes from the track endpoint saved after processing the previous image pair. After point-level matching is completed for the current image pair, the system checks whether the starting point of a candidate match in the shared frame $I_{k}$ falls near an existing historical track endpoint, thereby determining whether the candidate match inherits the original internal track ID.

For the current image pair $\left(I_{k},I_{k+1}\right)$, A2PM first generates candidate point-level matches under region constraints. The *j*-th candidate match is denoted as:

(2) $$m_{j}=\left(p_{k,j}^{0},p_{k+1,j}^{1}\right),$$

where the subscript *k* indicates that the point is in the shared frame $I_{k}$ of the current image pair, and the subscript *k* + 1 indicates that the point is in the next frame $I_{k+1}$. The index *j* denotes the *j*-th candidate match in the current image pair. Superscripts 0 and 1 are used to distinguish the starting point and endpoint of the candidate match in the image pair. Therefore, $p_{k,j}^{0}$ denotes the starting point of the *j*-th candidate match in the shared frame $I_{k}$, and $p_{k+1,j}^{1}$ denotes its endpoint in the next frame $I_{k+1}$. Then, the system constructs a neighborhood gate $B\left(p^{\prime },r\right)$ centered at $p^{\prime }$ with radius r and determines in the shared frame $I_{k}$ whether the candidate starting point can be associated with an existing historical track. Here, r is a pixel-distance threshold used to determine whether the current candidate starting point lies within the neighborhood of a historical track endpoint. The inheritance condition is:

(3) $$||p_{k,j}^{0}-p^{\prime }|{|}_{2}<r$$

**Figure 3:**
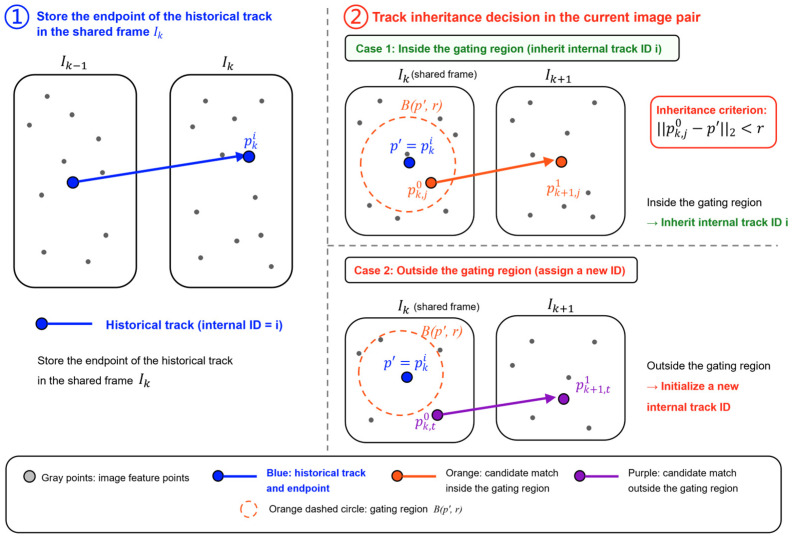
Illustration of the historical track inheritance mechanism across consecutive frame pairs. Taking three consecutive frames $\left(I_{k-1},I_{k},I_{k+1}\right)$ as an example, after matching the previous image pair $\left(I_{k-1},I_{k}\right)$, the system stores the historical track endpoint $p_{k}^{i}$ in the shared frame $I_{k}$ and uses it as the track inheritance reference for the current image pair $\left(I_{k},I_{k+1}\right)$. If the starting point of a candidate match falls inside the gating region $B\left(p^{\prime },r\right)$, it inherits the internal track ID; otherwise, it is initialized as a new internal track. The meanings of the gray points, blue track, orange match, and purple match are given in the legend.

When the above condition holds, the candidate match $m_{j}$ is regarded as the continuation of the *i*-th historical track in the current image pair and inherits its internal track ID. Then, the endpoint $p_{k+1,j}^{1}$ of the candidate match is saved as the historical endpoint of this track for association in the next image pair. For candidate matches that cannot be associated with an existing historical track but are still written into the current continuous matching result, the system initializes them as new internal tracks and assigns new internal track IDs. Candidate matches that are not retained do not enter the subsequent continuous matching file and do not participate in back-end optimization. The inheritance process is shown in Figure 3.

It should be emphasized that the internal track ID is only used in the A2PM preprocessing stage to maintain continuous matching relationships across adjacent frame pairs. Its role is to organize originally discrete pairwise matching results into more continuous feature tracks. The matching file finally output to the VINS-Fusion back end still follows the standard pairwise match format, and the back end does not directly read this internal track ID. In this way, A2PM-VINS allows matching results across consecutive frame pairs to be extended into feature tracks more stably without changing the back-end interface of VINS-Fusion, thereby reducing the effect of track fragmentation on back-end optimization.

### 2.3. Anchor–Explorer Feature Selection

In nonlinear optimization, the number of features is not linearly correlated with the final estimation accuracy. Deep networks usually generate a large number of candidate feature points, and excessive redundant features, especially those with low parallax or short lifetimes, not only increase computational cost but also dilute the effect of high-quality constraints in optimization, thereby limiting estimation accuracy. To address this issue, this paper proposes an Anchor–Explorer (A–E) dual-role feature selection mechanism to adaptively construct a feature subset that balances “stability” and “geometric contribution” from the candidate features. This mechanism divides candidate features into two complementary categories and scores them separately.

Among them, Anchor features are mainly used to provide stable geometric constraints so as to suppress trajectory drift. Therefore, long tracking lifetime and high geometric consistency are prioritized during selection. The scoring function $S_{Anchor}\left(k\right)$ is defined as follows:

(4) $$S_{Anchor}\left(k\right)=\left[w_{geo}\cdot G\left(k\right)+w_{age}\cdot T\left(k\right)\right]\cdot R\left(k\right)\cdot B\left(k\right)$$

Here, the term in brackets is used to measure the basic geometric quality of the feature, while the latter two terms are used to further suppress the influence of unreliable features. The geometric-consistency term $G\left(k\right)$ is defined as follows:

(5) $$G\left(k\right)=1/\left(1+d_{sampson}\left(k\right)/\sigma_{s}\right),$$

where $d_{sampson}\left(k\right)$ denotes the Sampson distance of feature *k*, and $\sigma_{s}$ denotes the normalization scale factor. $T\left(k\right)$ denotes the normalized track age and is used to measure the survival time or tracking length of the feature, thereby rewarding long-lived features that have been tracked stably. $R\left(k\right)$ denotes the reprojection-consistency term, which evaluates the residual by using the Tukey kernel function so as to truncate outliers. $B\left(k\right)$ denotes the boundary-distance term, which penalizes features close to the image boundary in order to reduce track interruption caused by camera motion. The coefficients $w_{geo}$ and $w_{age}$ represent the weights of the geometric-consistency term and the track-age term, respectively, and are used to balance their contributions to the Anchor score.

Correspondingly, Explorer features are mainly used to enhance geometric contribution and accelerate depth convergence. To capture features with high dynamics or rich structure, the scoring function $S_{Explorer}(k)$ is defined as follows:

(6) $$S_{Explorer}(k)=w_{par}\cdot M_{motion}(k)+w_{struct}\cdot M_{struct}(k)$$

where $w_{par}$ and $w_{struct}$ control the relative contributions of the parallax-gain term and the structural-information term, respectively. The motion-gain term $M_{motion}(k)$ is defined as follows:

(7) $$M_{motion}\left(k\right)=P\left(k\right)\cdot \beta \left(k\right)$$

This term explicitly prioritizes features with significant parallax by using the normalized parallax $P\left(k\right)$ together with the nonlinear boosting factor $\beta \left(k\right)$. $M_{struct}(k)$ denotes the structural-gain term and is used to reward features whose motion direction is orthogonal to the dominant optical-flow direction, which can provide stronger rotational degree-of-freedom constraints, as well as features located in high-gradient regions.

After defining the above two complementary scoring functions, this paper further designs a spatially uniform dual-channel selection algorithm in order to avoid the tendency of conventional methods to cluster a large number of features in highly textured regions while neglecting weak-texture regions, and to ensure greater diversity in the geometric information provided by the selected features. Specifically, the current image is divided into K non-overlapping grid cells, with a grid size of 40 × 40 pixels. For each grid cell $G_{M}$, the system constructs a local feature subset $F_{select}^{\left(m\right)}$ by using a dual-channel selection strategy. First, the feature $f_{a}^{*}$ with the highest $S_{Anchor}$ score in that grid is searched for and preserved as the stable anchor point selected by the primary channel to maintain the basic pose constraints. Then, after excluding $f_{a}^{*}$, the feature $f_{e}^{*}$ with the highest $S_{Explorer}$ score is searched for among the remaining candidates and preserved as the complementary feature selected by the secondary channel so as to retain the additional geometric information brought by large parallax or non-collinear motion. The local selection process can be written as follows:

(8) $$F_{select}^{\left(m\right)}=\{f_{a}^{*}|f_{a}^{*}=\underset{k\in G_{m}}{arg\;max}\;S_{Anchor}(k)\}\cup \{f_{e}^{*}|f_{e}^{*}=\underset{k\in G_{m},k\neq f_{a}^{*}}{arg\;max}\;S_{Explorer}(k)\}$$

**Figure 4:**
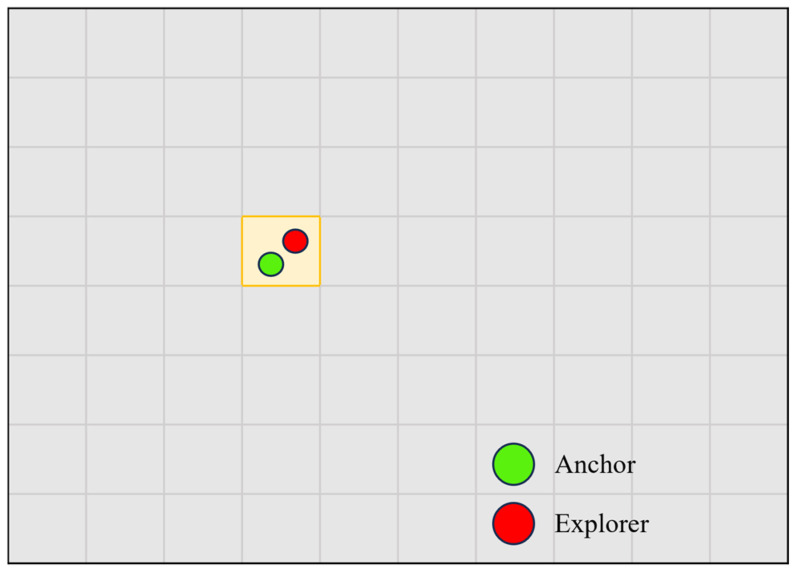
Illustration of the Anchor–Explorer (A–E) dual-role feature selection mechanism. The image is divided into multiple 40 × 40 pixel grid cells. In each grid, one Anchor feature (green point) and one Explorer feature (red point) are selected according to the Anchor score and the Explorer score, respectively, so that long-term stable constraints and high-parallax features are jointly preserved while maintaining a uniform spatial distribution. At a resolution of 752 × 480, 450 denotes the nominal upper feature budget in the A–E feature selection stage, rather than a fixed number of features retained in every frame. The actual number of features entering the back end is also affected by the distribution of candidate matches, the number of valid candidates in each grid, and the supplementary retention process. Therefore, the later experiments in this paper analyze the actual average number of retained features obtained from logs.

Through the above strategy, the system achieves both spatial uniformity of feature distribution and diversity of constraint information while keeping the total number of features under control, thereby reducing the adverse influence of local feature over-concentration and the resulting information redundancy on back-end optimization. The grid-based Anchor–Explorer feature selection result is illustrated in Figure 4.

### 2.4. SWC-Based Back-End Optimization

**Figure 5:**
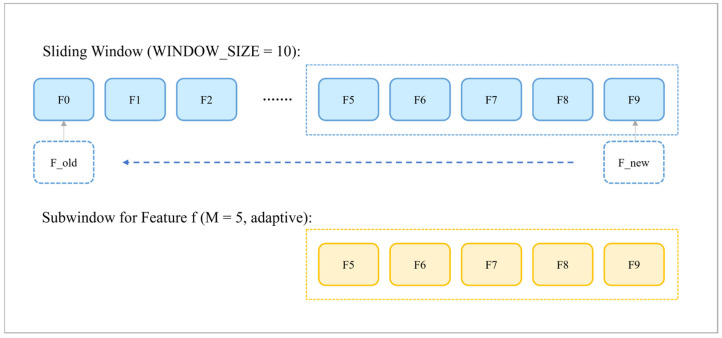
Illustration of the relationship between the fixed sliding window and the feature-adaptive sub-window in Sub-Window Consistency (SWC). F0–F9 denote the ten consecutive frames maintained in the fixed sliding window, where WINDOW_SIZE = 10. The upper row represents the fixed sliding-window timeline, and the blue boxes highlight the frames that correspond to the SWC sub-window shown below. The lower row represents the SWC sub-window view, where the yellow boxes correspond one-to-one to the blue boxes with the same frame indices. In this example, F5–F9 form a five-frame SWC sub-window for consistency-based weighting. In the actual implementation, the SWC sub-window length is adjusted between 3 and 5 frames according to the available observations.

The back end of A2PM-VINS follows the sliding-window optimization framework of VINS-Fusion. To maintain computational efficiency while preserving estimation accuracy, the system performs local optimization within a fixed-size sliding window. In this paper, WINDOW_SIZE = 10, meaning that the system always maintains the most recent 10 state nodes and their associated visual landmarks. Figure 5 shows the relationship between the fixed sliding window and the feature-adaptive sub-window. Within this back-end framework, the system jointly uses visual reprojection constraints, IMU preintegration constraints, and marginalization priors for state estimation. In addition to the robust kernel function, this paper further introduces a dynamic weighting mechanism based on Sub-Window Consistency (SWC) for visual residuals so as to suppress geometrically deceptive observations that may exhibit low reprojection errors in repetitive-texture environments.

Formally, the sensor-fusion problem within the sliding window can be formulated as a maximum a posteriori estimation problem. In the optimization variable X, the system jointly estimates the IMU states at different time instants within the sliding window and the inverse-depth parameters of visual landmarks. The IMU state of a single frame consists of position, orientation, velocity, and the biases of the accelerometer and gyroscope. Visual landmarks are parameterized by inverse depth so as to improve the numerical stability of distant features during optimization. The overall objective function is written as follows:

(9) $${min}_{X}\left\{\underset{Marginalization\;Prior}{\underbrace{∥r_{p}-H_{p}X{∥}^{2}}}+\underset{IMU\;Pre-integration\;Constraint}{\underbrace{\sum_{k\in B}∥r_{B}\left({\hat{z}}_{b_{k+1}}^{B},x_{k},x_{k+1}\right){∥}_{P_{b_{k+1}}^{B}}^{2}}}+\underset{Weighted\;Visual\;Reprojection}{\underbrace{\sum_{\left(i,j\right)\in F}\omega_{i,j}^{SWC}\cdot \rho \left(∥r_{C}\left({\hat{z}}_{i,j}^{C},X\right){∥}_{P_{i,j}^{C}}^{2}\right)}}\right\}$$

Here, the first term denotes the marginalization prior residual, the second term denotes the IMU preintegration residual, and the third term denotes the visual reprojection residual dynamically weighted by SWC. The core modification of this work lies in introducing SWC-based dynamic weights into the visual reprojection residual term so as to suppress geometrically deceptive observations caused by repetitive textures.

In repetitive-texture environments, mismatches may present low reprojection errors in a single frame and therefore bypass simple frame-wise checks. To address this issue, the SWC mechanism retrospectively analyzes the temporal continuity and geometric consistency of tracks within the most recent sub-window so as to evaluate the reliability of each feature track. It should be noted that the SWC sub-window length is not a fixed constant but adaptively varies between 3 and 5. For tracks with short observation histories, the sub-window length can be shortened to 3. For tracks with sufficient continuous observations, it can be extended up to 5. For an arbitrary track, the SWC weight $\omega_{\tau }^{SWC}$ is defined as follows:

(10) $$\omega_{\tau }^{SWC}=C_{temp}(\tau )\times C_{geom}(\tau )={\left(\frac{L_{\tau }}{M_{sub}}\right)}^{\gamma }\times (\frac{1}{1+\sigma_{epi}\cdot \Phi_{TSS}+\epsilon })$$

where *τ* denotes the current feature track, $C_{temp}(\tau )$ denotes the temporal continuity term, and $C_{geom}(\tau )$ denotes the geometric consistency term. $L_{\tau }$ denotes the number of valid observations of track *τ* within the sub-window, $M_{sub}$ denotes the current sub-window length, γ denotes the temporal continuity decay coefficient, $\sigma_{epi}$ denotes the standard deviation of the epipolar error, $\Phi_{TSS}$ denotes the triangulation stability score, and ϵ is a small constant introduced to prevent division by zero.

Mechanistically, the SWC weight is jointly determined by the temporal term and the geometric term. First, the temporal continuity term is used to evaluate the continuity of track observations within a local temporal window. A reliable feature track should usually maintain relatively stable continuous observations over the most recent frames. If the proportion of valid observations in the sub-window is low, this indicates that the track is obviously discontinuous, and its weight will be further attenuated by the temporal term. Second, the geometric consistency term is used to evaluate whether the observation is geometrically deceptive. This term couples the standard deviation of the epipolar error with the triangulation stability score. The underlying rationale is that a geometrically reliable landmark should simultaneously satisfy stable depth estimation and consistent epipolar geometry across multiple views. In contrast, incorrect matches caused by repetitive textures may sometimes show small depth variance, but because the observations from different viewpoints are inconsistent, they usually produce larger epipolar errors. The coupled term in the denominator can further amplify this difference, thereby reducing the influence of geometrically deceptive observations during optimization.

In implementation, the SWC statistics directly act on the weighting process of visual residual terms. Observations with higher consistency retain larger weights in the current round of optimization, whereas observations with poor continuity, fragmented tracks, or geometric instability are explicitly downweighted. Through this continuous weighting mechanism, back-end optimization relies more on observations with stronger temporal continuity and geometric consistency, thereby improving the stability of state estimation in degraded segments.

### 2.5. Experimental Setup

To validate the effectiveness of the proposed method, this paper conducts a systematic evaluation on the public European Robotics Challenge Micro Aerial Vehicle (EuRoC MAV) dataset [23]. This dataset contains two representative types of indoor scenes, namely Machine Hall (MH) and Vicon Room (V). The former contains a large number of regularly arranged structures and locally repetitive appearances, whereas the latter contains weak-texture regions and motion blur caused by aggressive maneuvers. Therefore, this dataset is suitable for evaluating localization performance in complex degraded environments.

The experimental hardware platform is equipped with an Intel Xeon-6154 processor, 128 GB of memory, and an NVIDIA K80 graphics processing unit, and the software environment is based on Ubuntu 18.04 and Robot Operating System (ROS) Noetic. In the proposed A2PM-VINS system, the back-end sliding-window size is fixed at 10 frames, the input images are uniformly resized to 752 × 480 pixels, the Anchor–Explorer grid size is set to 40 × 40 pixels, and the number of features participating in back-end optimization per frame is controlled within 450. Absolute Trajectory Error (ATE) and Relative Pose Error (RPE), including rotational RPE (RPEr) and translational RPE (RPEt) are adopted as the main evaluation metrics, and trajectory post-processing and statistical analysis are uniformly carried out using the evo tool [24].

The current implementation adopts a two-stage operating mode, namely “offline Area-to-Point matching preprocessing + online VINS state estimation”. In the offline stage, a text file of matching results is generated for each pair of adjacent image frames, and the pixel coordinates of the corresponding matched point pairs are recorded. In the online stage, VINS-Fusion reads the precomputed matching results and completes system state estimation by combining IMU preintegration, sliding-window optimization, loop closure detection, and global pose graph optimization.

In the specific implementation, SAM is used for region segmentation, DMESA is used for region-level matching, and LoFTR is used for point-wise matching within paired regions [9]. The back-end optimization framework is based on VINS-Fusion and retains its integrated DBoW2 loop closure detection and pose graph optimization module [25]. Trajectory evaluation is uniformly completed using the evo tool.

To make the attribution of subsequent ablation experiments clearer, this paper further distinguishes external system baselines from internal ablation baselines. VINS-Fusion and SuperVINS are used as external system baselines for the main trajectory-accuracy comparison. A2PM-VINS-CG is used as an internal conventional grid-based selection baseline to isolate the contribution of the Anchor–Explorer feature selection mechanism. A2PM-VINS-CG and A2PM-VINS use the same A2PM area-to-point matching results, historical track inheritance mechanism, and back-end optimization settings; only the Anchor–Explorer dual-role selection is replaced by conventional grid-based selection. The default A2PM-VINS main system in this paper includes A2PM area-to-point hierarchical matching, the historical track inheritance mechanism, and A–E feature selection, but does not include SWC. SWC is analyzed as an additional back-end enhancement module in a separate ablation experiment.

## 3. Results

### 3.1. Trajectory Accuracy

To evaluate the localization performance of A2PM-VINS from both global trajectory deviation and local relative motion error, this paper uses absolute trajectory error (ATE) and relative pose error (RPEr and RPEt) as the main metrics. This section mainly presents the overall accuracy differences among different methods on typical difficult sequences. The locations of local deviations and their relationship with front-end feature behavior are further shown in Section 3.2 using visualization results. First, ATE is used to evaluate the global trajectory error of different methods on the EuRoC sequences, and the results are shown in Table 1.

From the ATE results, A2PM-VINS shows superior or competitive global trajectory accuracy on multiple challenging sequences. For MH_04 and MH_05 in the Machine Hall scene, the ATE values of A2PM-VINS are 0.0983 and 0.1191, respectively, both of which are better than those of VINS-Fusion (Loop) and SuperVINS. In particular, the error reduction on MH_04 is more pronounced compared with VINS-Fusion (Loop), and a stable advantage is also maintained on MH_05. This indicates that, in challenging scenes with more evident low illumination and repetitive textures, A2PM-VINS can still achieve favorable global trajectory accuracy when compared with baseline methods with loop closure enabled.

In the Vicon Room scene, A2PM-VINS also demonstrates strong competitiveness on several difficult sequences. Taking V1_02 as an example, the ATE of A2PM-VINS is 0.1076, which is better than that of VINS-Fusion (Loop) and SuperVINS. On V2_02, VINS-Fusion fails to maintain stable tracking with or without loop closure, whereas A2PM-VINS still achieves an ATE of 0.1349, indicating that it can maintain stable tracking under extremely degraded conditions. For V2_03, the ATE of A2PM-VINS is better than that of VINS-Fusion (NoLoop), but worse than that of VINS-Fusion (Loop) and SuperVINS. On relatively simple sequences such as V1_01 and V2_01, A2PM-VINS does not show an advantage over the baseline methods, indicating that the benefits of the proposed method are mainly concentrated in complex degraded environments rather than in simple scenes.

Because Table 1 reports both the Loop and NoLoop results of VINS-Fusion, readers can more intuitively compare the influence of loop optimization on the global accuracy of the baseline system. More importantly for this paper, under the complete system configuration, A2PM-VINS achieves superior or competitive results on multiple challenging sequences, which is consistent with the joint design of region-constrained matching, temporal inheritance, and A–E dual-role selection.

Based on the above baseline comparison, to further improve the performance benchmarking of A2PM-VINS against recent visual–inertial SLAM methods, this paper selects DeepLine-VIO and XR-VIO as recent VIO methods for an additional ATE comparison. The results are shown in Table 2.

As shown in Table 2, A2PM-VINS obtains the lowest ATE on the MH_03, MH_04, and MH_05 sequences in the Machine Hall scenario. This indicates that the proposed method is competitive in MH scenes that contain many industrial structures, local repetitive textures, and degraded visual factors. Compared with DeepLine-VIO, A2PM-VINS obtains lower errors on most test sequences except V2_02, suggesting that region-constrained matching and A–E feature selection have a positive effect on trajectory estimation in complex-texture scenes.

At the same time, XR-VIO performs better on the V1_02, V1_03, V2_02, and V2_03 sequences in the Vicon Room scenario, especially on V1_02 and V2_02, where it achieves lower ATE values. This indicates that A2PM-VINS is not better than recent VIO methods on all sequences. Its advantage is mainly concentrated in the repetitive-texture and locally degraded visual scenes considered in this paper.

After completing the ATE comparison, this paper further evaluates the short-term estimation stability of different methods from the perspective of local relative motion error. The related RPE results are shown in Table 3.

The RPE results are generally consistent with the above ATE conclusions, indicating that the advantages of A2PM-VINS are reflected not only in accumulated global trajectory error but also in the stability of short-term local motion estimation. On the two challenging sequences MH_04 and MH_05, the RPEr values of A2PM-VINS are 0.0019 and 0.0031, respectively, both better than 0.0030 and 0.0034 of VINS-Fusion. Its translational errors RPEt are 0.0073 and 0.0082, respectively, both lower than 0.0098 and 0.0135 of VINS-Fusion, and also better than 0.0096 and 0.0095 of SuperVINS on these two sequences. This indicates that, in challenging scenes with repetitive textures and low illumination, the proposed method performs well in both global trajectory deviation and local motion estimation.

In the Vicon Room sequences, the improvement in local accuracy of A2PM-VINS is also evident. For example, on V1_02, its RPEt is 0.0052, which is significantly better than 0.0082 of VINS-Fusion and 0.0204 of SuperVINS. On V2_03, the RPEr and RPEt of A2PM-VINS are 0.0053 and 0.0082, respectively, both better than those of VINS-Fusion and SuperVINS. On V2_02, VINS-Fusion cannot provide RPE results due to tracking failure, whereas A2PM-VINS still achieves an RPEr of 0.0069 and an RPEt of 0.0081. This indicates that, even under extremely degraded conditions, A2PM-VINS can still maintain stable tracking and complete local motion estimation. Taken together, the ATE and RPE results suggest that A2PM-VINS achieves favorable overall performance on multiple challenging sequences in terms of both global consistency and local motion stability.

### 3.2. Trajectory and Feature Visualization

**Figure 6:**
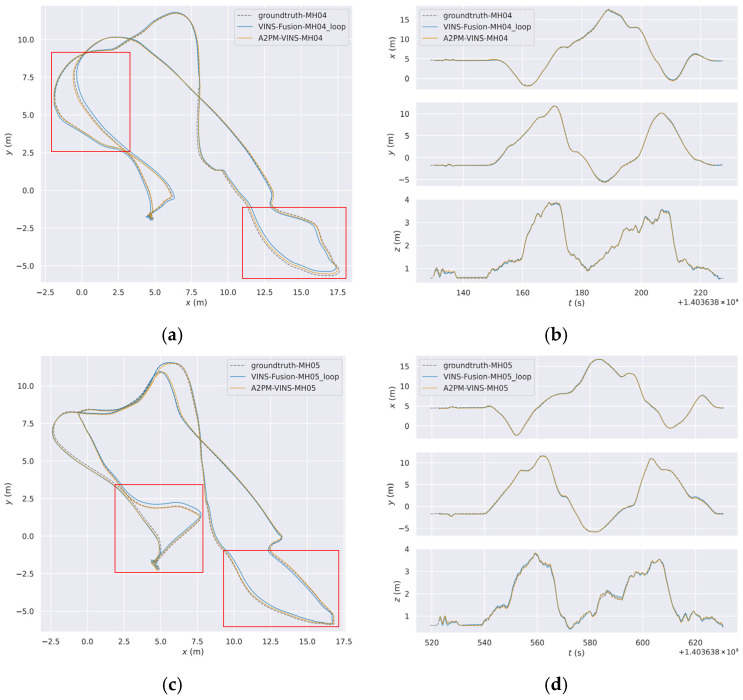
Comparison of global trajectories and axis-wise temporal trajectories on the MH_04 and MH_05 sequences. (**a**) Comparison of global trajectories on the MH_04 sequence; (**b**) comparison of temporal trajectories along the x, y, and z axes on the MH_04 sequence; (**c**) comparison of global trajectories on the MH_05 sequence; (**d**) comparison of temporal trajectories along the x, y, and z axes on the MH_05 sequence. The red rectangles mark representative segments selected for subsequent local trajectory zoom-in analysis, which are used to show the trajectory differences of different methods in local difficult regions.

**Figure 7:**
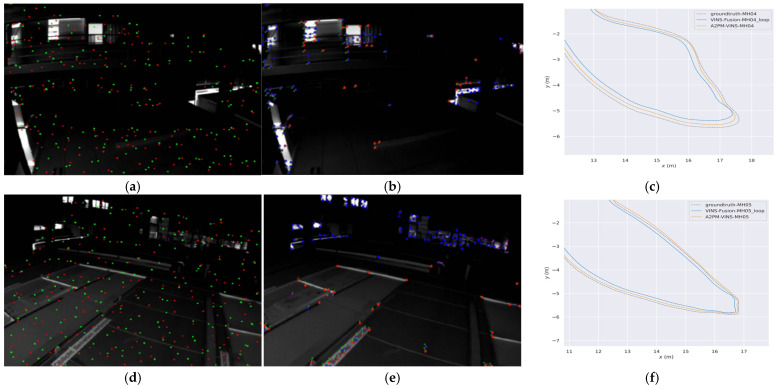
Comparison of feature distributions and local trajectories in low-light scenes with repetitive block-like ground textures. (**a**) Front-end feature distribution of A2PM-VINS in a local challenging segment of MH_04; (**b**) front-end feature distribution of VINS-Fusion in a local challenging segment of MH_04; (**c**) enlarged comparison of trajectories in the corresponding local segment of MH_04; (**d**) front-end feature distribution of A2PM-VINS in a local challenging segment of MH_05; (**e**) front-end feature distribution of VINS-Fusion in a local challenging segment of MH_05; (**f**) enlarged comparison of trajectories in the corresponding local segment of MH_05. In (**a**,**d**), the green and red dots correspond to the Anchor and Explorer features defined in the Anchor–Explorer feature selection mechanism, respectively. In (**b**,**e**), the colored dots indicate feature points visualized by VINS-Fusion.

To more intuitively analyze the behavioral differences among different methods in complex degraded scenes, this subsection provides three groups of visual results. Figure 6 presents the global trajectories and axis-wise temporal trajectories of MH_04 and MH_05, which are used to compare the trajectory fitting degree between different methods and the ground truth from an overall perspective. Figure 6 and Figure 7 further combine real local scenes, front-end feature-point distributions, and enlarged local trajectory results to illustrate the differences among methods on typical degraded segments. This organization helps directly associate local trajectory deviation with the corresponding real degraded scene, thereby providing a more concrete explanation of the relationship between front-end feature behavior and trajectory results.

From Figure 6, it can be seen that on the two challenging sequences MH_04 and MH_05, the global trajectories of A2PM-VINS fit the ground-truth trajectories better overall. Compared with VINS-Fusion, A2PM-VINS shows smaller local deviations on several key segments. In the axis-wise temporal trajectories, its variation trends along the x, y, and z directions are also more consistent with the ground truth. This indicates that the proposed method offers advantages in both global trajectory consistency and local motion-estimation stability.

**Figure 8:**
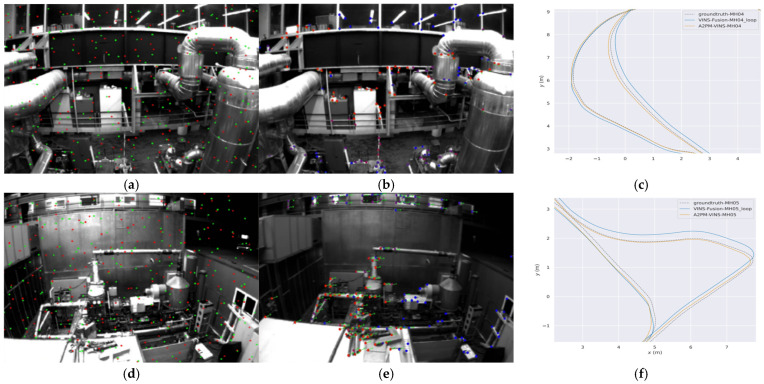
Comparison of feature distributions and local trajectories in scenes with regular glass windows and repetitive block-like box structures. (**a**) Front-end feature distribution of A2PM-VINS in a local challenging segment of MH_04; (**b**) front-end feature distribution of VINS-Fusion in a local challenging segment of MH_04; (**c**) enlarged comparison of trajectories in the corresponding local segment of MH_04; (**d**) front-end feature distribution of A2PM-VINS in a local challenging segment of MH_05; (**e**) front-end feature distribution of VINS-Fusion in a local challenging segment of MH_05; (**f**) enlarged comparison of trajectories in the corresponding local segment of MH_05. In (**a**,**d**), the green and red dots correspond to the Anchor and Explorer features defined in the Anchor–Explorer feature selection mechanism, respectively. In (**b**,**e**), the colored dots indicate feature points visualized by VINS-Fusion.

Figure 7 and Figure 8 further present the real scenes, front-end feature distributions, and enlarged local trajectory results on typical local difficult segments. It can be observed that in degraded scenes such as low illumination, repetitive block-like ground textures, regular glass windows, and repetitive box-like structures, the feature points retained by A2PM-VINS are more uniformly distributed. In contrast, VINS-Fusion is more likely to exhibit feature sparsity or local clustering. Correspondingly, in the local trajectory plots on the right, the trajectories of A2PM-VINS generally fit the ground truth better, whereas the deviation of VINS-Fusion is more obvious. This indicates that the joint design of the A2PM front end, temporal inheritance, and feature selection helps improve front-end feature distribution and tracking statistics, and corresponds to better trajectory fitting on local difficult segments.

In addition, the feature-point distributions in Figure 7 and Figure 8 also reflect the influence of the A–E dual-role feature selection mechanism on front-end feature distribution and tracking statistics. Compared with VINS-Fusion, the feature points retained by A2PM-VINS are more uniformly distributed in space and show less concentration in locally highly textured regions. This, to some extent, reflects the effect of the A–E mechanism on front-end feature distribution and tracking statistics, indicating that this mechanism helps improve the feature distribution entering the back end.

It should be emphasized that the advantage here is not reflected in simply increasing the number of features, but rather in the fact that the retained features are more beneficial to back-end optimization in terms of spatial distribution and geometric consistency. By presenting the real local scene, feature distribution, and local trajectory results in the same set of figures, it can be seen that the advantage of A2PM-VINS in complex degraded scenes is related to the improvement of front-end feature quality and its influence on back-end optimization.

### 3.3. Analysis of Front-End Feature Mechanisms

To quantitatively analyze the changes in front-end feature distribution and tracking statistics of A2PM-VINS, Table 4 presents the comparison of average front-end feature-related statistics on the V1_02 sequence. V1_02 is selected here as a detailed quantitative example because this sequence allows both VINS-Fusion and A2PM-VINS to run stably, thus making it easier to clearly show the differences in front-end feature distribution and continuous tracking statistics.

The results show that A2PM-VINS retains an average of 323.97 current-frame feature points on this sequence, which is significantly higher than the 150.00 of VINS-Fusion. Its average number of continuously tracked points increases from 103.55 to 232.51, and the average continuous tracking ratio also rises from 0.6904 to 0.7184. Although the average track length decreases from 8.22 to 6.43, the overall results indicate that A2PM-VINS achieves a better balance between observation coverage and short-term continuous tracking capability. This suggests that the benefit of the method does not rely on simply increasing the number of features, but is related to the improvement in feature distribution and short-term continuous tracking capability.

To further isolate the contribution of the Anchor–Explorer (A–E) dual-role feature selection mechanism itself, this paper adds an A–E ablation experiment. It should be noted that A2PM-VINS-CG is neither the original VINS-Fusion nor a version without feature selection. Instead, it is an internal conventional grid-based selection baseline under A2PM candidate matching results. This baseline uses the same area-to-point matching results, historical track inheritance mechanism, and back-end optimization parameters as A2PM-VINS-AE, and SWC is disabled in both cases. The only difference lies in the candidate feature selection strategy: A2PM-VINS-CG uses conventional grid-based selection, while A2PM-VINS-AE uses the Anchor–Explorer dual-role scoring and selection mechanism. Table 5 gives the per-sequence ATE results of the A–E ablation experiment.

Table 5 first shows the effect of the A–E mechanism on trajectory accuracy from a per-sequence perspective. A2PM-VINS-AE reduces the ATE on all six test sequences. The average ATE decreases from 0.1597 m to 0.1303 m, corresponding to a reduction of about 18.4% based on the average ATE. The largest reduction is observed on V2_03, where the ATE decreases from 0.2803 m to 0.2009 m. This indicates that the benefit of the A–E mechanism is not limited to a single sequence, but appears consistently across several scenes with degraded factors. Table 6 further analyzes the A–E mechanism from three aspects: feature quality, back-end optimization, and computational load.

Table 6 shows that, compared with A2PM-VINS-CG, A2PM-VINS-AE increases the average number of retained features from 302.5 to 337.8, but the continuous tracking ratio, average track length, and long-track ratio are not higher than those of CG. This indicates that A–E does not simply extend feature lifetime or pursue a higher continuous tracking ratio. Instead, it selects an observation set that is more suitable for back-end optimization by combining Anchor stability and Explorer geometric supplementation.

From the back-end optimization process, A–E reduces the average number of Ceres iterations from 7.26 to 6.32, suggesting that the selected feature set helps improve the convergence process of sliding-window optimization. However, in terms of computational load, A–E introduces additional front-end selection cost. The selection time increases from 0.29 ms to 9.52 ms, and the solver time also slightly increases from 34.05 ms to 36.21 ms. Therefore, this paper does not describe A–E as a module for reducing computational cost. Instead, it is positioned as a front-end feature selection mechanism that trades a certain selection cost for a higher-quality observation set and lower trajectory error.

To analyze the sensitivity of A–E to the grid-size parameter, this paper changes only the grid size while keeping the A–E dual-role selection mechanism unchanged, and reports the ATE results on each test sequence in Table 7. In this experiment, SWC remains disabled, and each grid still uses Anchor and Explorer dual-role selection.

**Figure 9:**
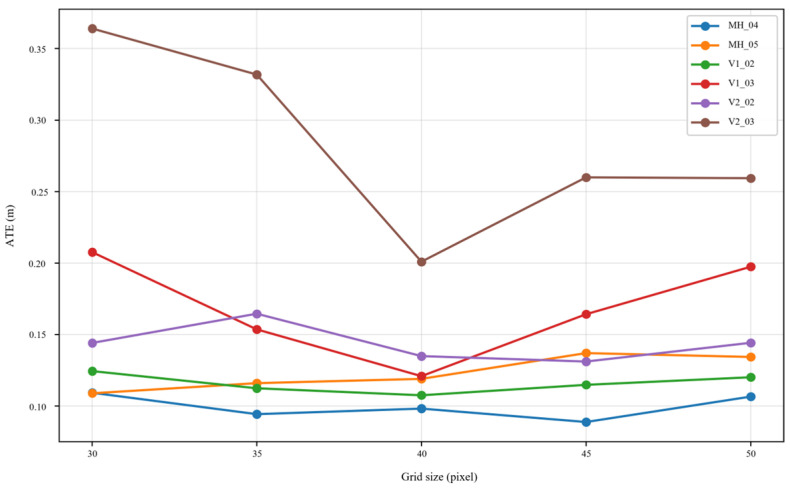
Absolute Trajectory Error (ATE) variation in EuRoC test sequences under different grid sizes. The Anchor–Explorer dual-role selection mechanism is kept unchanged, and only the grid size is varied. Different grid settings affect the ATE values of different sequences in different ways, but 40 × 40 achieves the lowest cross-sequence average ATE and shows relatively stable overall performance.

To show the ATE variation trend more intuitively under different grid sizes, Figure 9 gives the ATE curves for the six test sequences.

Table 7 and Figure 9 show that the effect of different grid settings on the ATE is not the same across sequences. On MH_04 and V1_02, the ATE variation under different grid settings is relatively small, indicating that grid-scale changes have a limited effect on these two results. On MH_05 and V2_02, the ATE shows moderate variation, and some neighboring grid settings obtain slightly lower errors. On V1_03 and V2_03, the ATE variation is larger, indicating that in more difficult visual conditions, the spatial partition scale of features more clearly affects the quality of observations entering back-end optimization.

Specifically, 40 × 40 obtains the lowest ATE on V1_02, V1_03, and V2_03; 45 × 45 is slightly better on MH_04 and V2_02; and 30 × 30 is slightly better on MH_05. Although 40 × 40 is not the best setting on every individual sequence, it achieves the lowest cross-sequence average ATE of 0.1303 m across the six sequences, indicating more stable overall performance across different scenes.

It should be noted that the grid size and the dual-role candidates in each grid determine the nominal capacity at the grid stage, rather than the exact final number of features entering the back end. Since the spatial distribution of A2PM candidate matches varies from frame to frame, the final number of retained features is determined by the complete A–E selection process. To further analyze this relationship, Table 8 summarizes the nominal capacity, actual average retained feature count, continuous tracking ratio, and computational cost under different grid sizes.

Table 8 further shows that, as the grid size increases from 30 × 30 to 50 × 50, the nominal grid-stage capacity decreases from 832 to 320, and the actual average number of retained features decreases from 500.4 to 299.2. The 30 × 30 grid retains the largest number of features, but its average ATE is 0.1765 m, which is higher than that of the default 40 × 40 setting. This suggests that more features are not necessarily better, and overly dense grids may introduce more local redundant observations. On the other hand, the 50 × 50 grid has the highest continuous tracking ratio of 0.777, but its average ATE is 0.1604 m and is not the best. This indicates that a higher continuous tracking ratio does not necessarily correspond to lower trajectory error. Overall, 40 × 40 provides a reasonable trade-off among actual retained feature count, spatial coverage, redundancy control, and trajectory accuracy, and is therefore retained as the default grid setting in this paper.

### 3.4. SWC Ablation

To analyze the actual contribution of the Sub-Window Consistency (SWC) module to system performance, this paper further conducts ablation experiments on two typical challenging sequences, MH_04 and MH_05, and compares the ATE changes of A2PM-VINS with and without SWC. The results are shown in Table 9.

**Figure 10:**
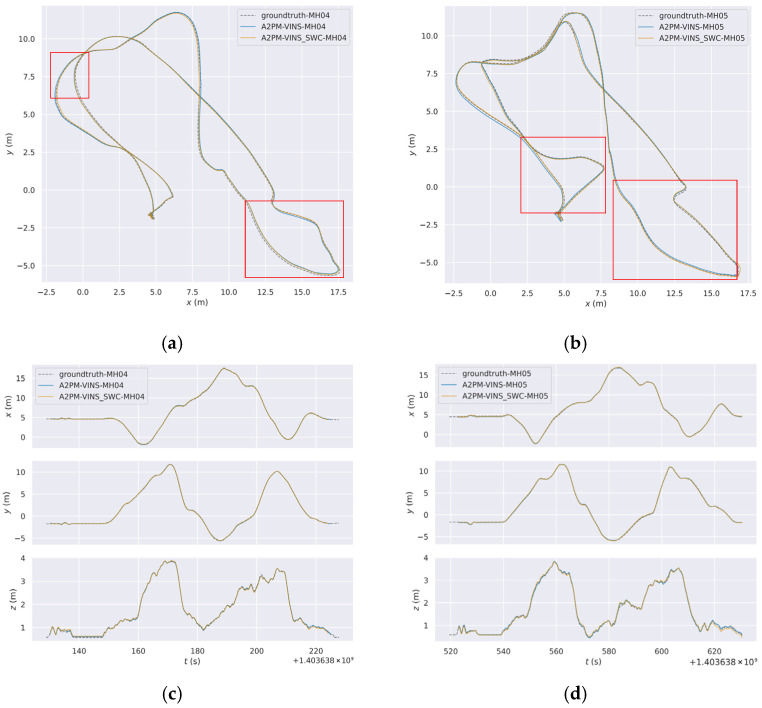
Global trajectory and x-, y-, and z-axis trajectory comparisons on the MH_04 and MH_05 sequences with and without SWC. (**a**) Global trajectory comparison between A2PM-VINS and A2PM-VINS + SWC on MH_04; (**b**) global trajectory comparison between A2PM-VINS and A2PM-VINS + SWC on MH_05; (**c**) x-, y-, and z-axis trajectory comparison on MH_04; (**d**) x-, y-, and z-axis trajectory comparison on MH_05.The red rectangles mark representative segments selected for local trajectory zoom-in analysis, which are used to observe the effect of SWC dynamic weighting on local trajectory deviation.

**Figure 11:**
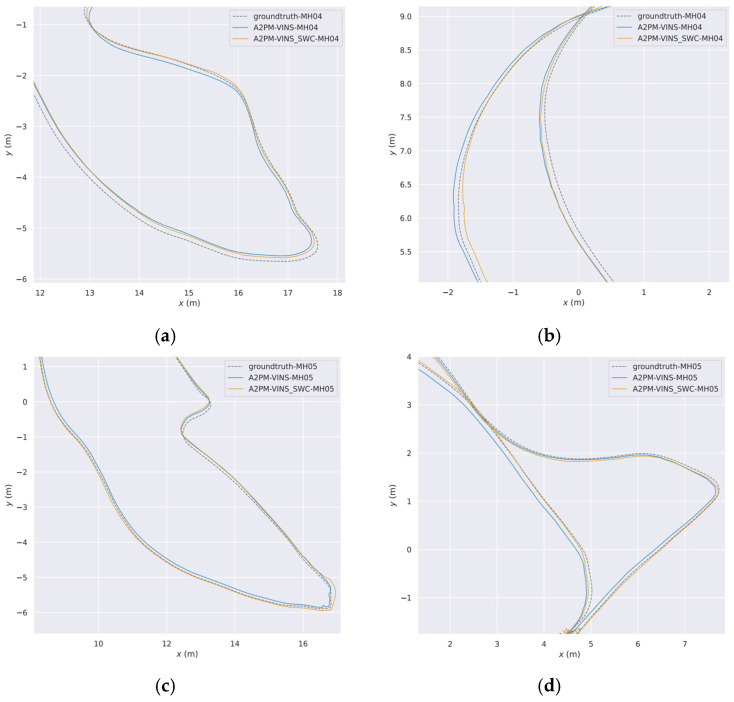
Enlarged comparison of trajectories in local challenging segments of MH_04 and MH_05 with and without SWC. (**a**) Enlarged comparison of trajectories in local challenging segment 1 of MH_04 with and without SWC; (**b**) enlarged comparison of trajectories in local challenging segment 2 of MH_04 with and without SWC; (**c**) enlarged comparison of trajectories in local challenging segment 1 of MH_05 with and without SWC; (**d**) enlarged comparison of trajectories in local challenging segment 2 of MH_05 with and without SWC.

Table 9 shows that SWC further reduces the ATE on MH_04, MH_05, and V1_02, with a relatively clear reduction on MH_05. Together with the local trajectory zoom-in results in Figure 10 and Figure 11, the results show that in the selected low-illumination, repetitive block-floor, and regular-structure segments, the local trajectory deviation is reduced after adding SWC. This indicates that SWC can provide auxiliary improvement in some low-illumination and repetitive-structure segments.

However, the benefit of SWC is not universal. On V1_03, V2_02, and V2_03, the ATE increases after SWC is enabled. Considering the scene characteristics of these sequences, SWC still has limitations in scenes with rapid motion, weak textures, or noticeable viewpoint changes. Its local consistency weighting does not always reduce the trajectory error of the whole sequence. Therefore, this paper does not include SWC as a default component of the main A2PM-VINS system, and its adaptability to different degraded scenes still needs to be improved in future work.

Figure 10 gives the global trajectory comparison and marked local zoom-in regions on the MH_04 and MH_05 sequences with and without SWC. To show the effect of SWC on local segments more clearly, Figure 11 further zooms in on the representative segments marked in Figure 10.

Figure 10 and Figure 11 show that, before and after adding SWC, the overall trajectory shapes of the two sequences remain consistent. However, on several local segments, the trajectory after adding SWC aligns better with the ground truth. In the illustrated local difficult segments of MH_04 and MH_05, the trajectory with SWC is generally closer to the ground truth, while the result without SWC shows more visible local deviation at the same locations. It should be noted that these local segments correspond to the typical difficult scenes shown earlier, so the scene images are not repeated here.

### 3.5. Computational Complexity and Operational Overhead

It should be noted that the offline preprocessing used in this paper is not adopted simply for implementation convenience, but is mainly limited by the experimental hardware platform and the complete processing pipeline. In the original A2PM-related work, the region matching method DMESA can reach about 700 ms on a specific platform, but this result mainly reflects the efficiency of the region matching module itself. In contrast, the computational resources of the experimental platform used in this paper are relatively limited. In addition, the offline preprocessing pipeline in this paper includes several steps, such as geometric region refinement, point-level match generation, continuity linking, and result saving. Therefore, the overall processing time is much longer. The average processing time of the complete adjacent-frame preprocessing pipeline is 63,073.25 ms, and the average time of the region matching stage is 18,853.86 ms.

From the online estimation stage, the back-end runtime of A2PM-VINS remains within an acceptable range, indicating that the main computational bottleneck of the current system lies in the offline front-end preprocessing stage rather than in the VINS-Fusion back end itself. In other words, the focus of this paper is to verify the effectiveness of region-constrained matching and feature selection in degraded environments, rather than to build a front-end system that can be directly deployed in real time.

Based on the above overall explanation, this paper further decomposes the runtime cost of A2PM area-to-point matching, the historical track inheritance mechanism, A–E feature selection, and SWC back-end dynamic weighting. The results are shown in Table 10. The runtime cost is divided into offline preprocessing and online estimation. Offline preprocessing includes SAM region segmentation, DMESA region matching, region-constrained point-level matching, and the track inheritance mechanism. Online estimation includes candidate feature selection, sliding-window solving, and SWC weight updating. It should be noted that Ceres solver time only denotes the pure solving time of the back-end nonlinear solver and is not equal to the complete back-end processing time.

Table 10 decomposes the runtime cost of the main components of the proposed method. The largest computational cost of the current system comes from offline preprocessing related to A2PM area-to-point matching. The complete adjacent-frame preprocessing pipeline takes 63,073.25 ms on average, and the DMESA region matching stage takes 18,853.86 ms on average. This part improves matching reliability in complex scenes, but also limits the real-time deployability of the current implementation. Therefore, this paper regards it as the main engineering bottleneck of the current system and further discusses in the conclusion that lightweight region extraction, parallel processing, and hardware acceleration may reduce this cost in future work.

The track inheritance mechanism mainly includes candidate match association between consecutive image pairs, tracklet ID inheritance, and track state maintenance, with an average time of 437.42 ms. In the online part, A–E introduces additional feature selection time compared with CG. The average selection time increases from 0.29 ms/frame to 9.52 ms/frame. This result indicates that the accuracy benefit of A–E does not come from lower front-end runtime, but from selecting more useful observations. SWC has only a small effect on the pure Ceres solver time. The average solver time is 36.21 ms without SWC and 36.22 ms with SWC. Therefore, the main limitation of SWC is not the additional solving cost, but the stability of its weighting effect across different scenes.

## 4. Conclusions

This paper proposes A2PM-VINS, a visual–inertial SLAM method based on area-to-point matching. Built on VINS-Fusion, the method introduces A2PM area-to-point hierarchical matching and a historical track inheritance mechanism in the front end to improve matching reliability and feature-track continuity in repetitive-texture and weak-texture scenes. It also designs an Anchor–Explorer dual-role feature selection mechanism to select observations that are more suitable for back-end optimization from candidate matches. In the back end, an SWC dynamic weighting strategy is further incorporated to suppress observations with poor temporal continuity and geometric consistency within local time windows.

Experiments on the EuRoC dataset show that A2PM-VINS achieves good trajectory accuracy and local trajectory alignment on several difficult sequences containing degraded factors such as low illumination, repetitive textures, and weak textures. Comparisons with VINS-Fusion, SuperVINS, and some recent VIO methods show that the proposed method is competitive in Machine Hall scenes with many regular structures and local repetitive textures. The A–E ablation experiment and grid-sensitivity analysis further show that feature selection for back-end optimization helps improve the quality of observations entering the sliding window. The SWC ablation experiment shows that this module can further reduce trajectory error on some sequences, but its effect is scene-dependent.

It should be noted that the current A2PM-VINS still has some limitations. Since region segmentation, region matching, and region-constrained point-level matching are performed through offline preprocessing, the main computational bottleneck of the system lies in the front-end A2PM matching pipeline, and the current system is not yet suitable for direct deployment in strict real-time scenarios. In its current form, the proposed pipeline is more suitable for offline or near-offline high-precision mapping, dataset-based analysis, and low-speed ground-robot inspection tasks where preprocessing latency is acceptable, rather than fast-moving UAV applications requiring strict real-time response. Future work will focus on reducing the computational bottleneck of the current offline front-end preprocessing stage. Improvements can be made from both hardware acceleration and software architecture optimization. On the one hand, lightweight region segmentation models, parallel region matching, candidate region caching, and point-level matching network acceleration can be explored to reduce the overall computational cost of the A2PM front end. On the other hand, C++/CUDA/TensorRT-based deployment can be further adopted to accelerate feature extraction, region matching, and point-level matching modules. In addition, the current point-level matching stage uses LoFTR as the matcher after region constraints. Future work can systematically compare different point-level matchers under the same region-level constraint framework, so as to further analyze the effect of point-level matcher selection on matching stability, track continuity, and system accuracy. Recent efficient illumination-robust SLAM systems such as AirSLAM show that efficient deployment of learning-based feature front ends and the use of point–line structural cues can improve the practicality and robustness of systems under complex illumination conditions. Inspired by this, future work may also incorporate illumination-robust feature representations or point–line structural cues to further improve the front-end stability of A2PM-VINS under short-term and long-term illumination changes and to move the system toward online deployment.

In addition, complex degraded environments in real applications may include transient environmental disturbances such as water splashes, hail, raindrops, dust, and short-term occlusions. These disturbances can produce transient textures, edges, or abnormal image blocks, and may affect the reliability of front-end matching and feature selection. Wiseman [26] showed that JPEG compression statistics and block-level image analysis can be used for real-time visual change analysis in traffic monitoring scenarios, and pointed out that environmental factors such as water splashes and hail may cause false alarms. Inspired by this, future work can explore introducing block-level anomaly detection, compression statistical features, or lightweight visual quality assessment mechanisms into the A2PM-VINS front end to identify, suppress, or downweight candidate matches in transient disturbance regions, thereby further improving the robustness of the system in broader complex degraded environments.

## Figures and Tables

**Table 1:** Comparison of Absolute Trajectory Error (ATE) results on European Robotics Challenge Micro Aerial Vehicle (EuRoC MAV) sequences (including Loop/NoLoop).

Sequences	OKVIS	VINS-Mono (NoLoop)	VINS-Mono (Loop)	VINS-Fusion (NoLoop)	VINS-Fusion (Loop)	SuperVINS	A2PM-VINS (Ours)
MH_03	0.2500	0.1950	**0.0658**	0.1203	0.0919	0.1806	0.1257
MH_04	0.2700	0.3470	0.2047	0.2836	0.1307	0.1708	**0.0983**
MH_05	0.3900	0.3020	0.1421	0.1587	0.2621	0.1583	**0.1191**
V1_01	0.0940	0.0889	**0.0484**	0.1569	0.1536	0.2010	0.1686
V1_02	0.1400	0.1105	**0.0631**	0.1222	0.2192	0.1160	0.1076
V1_03	0.2100	0.1875	0.2012	0.1287	0.1700	0.2214	**0.1210**
V2_01	0.0900	0.0863	0.0672	0.1207	0.1233	**0.0611**	0.1286
V2_02	0.1700	0.1580	0.1588	—	—	**0.1003**	0.1349
V2_03	0.2300	0.2775	0.2475	0.2881	0.1926	**0.1687**	0.2009

Note: bold values indicate the lowest ATE in each row.

**Table 2:** Additional Absolute Trajectory Error (ATE) comparison with recent visual–inertial odometry (VIO) methods on selected EuRoC sequences.

Sequences	DeepLine-VIO	XR-VIO	A2PM-VINS (Ours)
MH_03	0.225	0.164	**0.** **126**
MH_04	0.231	0.181	**0.** **098**
MH_05	0.280	0.215	**0.119**
V1_02	0.124	**0.041**	0.108
V1_03	0.147	**0.075**	0.121
V2_02	0.128	**0.081**	0.135
V2_03	0.213	**0.100**	0.201

Note: bold values indicate the lowest ATE in each row.

**Table 3:** Comparison of Relative Pose Error (RPE) results on EuRoC sequences.

Sequences	RPE_r (Fusion)	RPE_r (SuperVINS)	RPE_r (A2PM)	RPE_t (Fusion)	RPE_t (SuperVINS)	RPE_t (A2PM)
MH_03	0.0052	0.0017	**0.0014**	0.0096	0.0082	**0.0044**
MH_04	0.0030	0.0020	**0.0019**	0.0098	0.0096	**0.0073**
MH_05	0.0034	0.0038	**0.0031**	0.0135	0.0095	0.0082
V1_01	0.0043	**0.0021**	0.0026	**0.0074**	0.0080	0.0083
V1_02	0.0042	**0.0027**	0.0033	0.0082	0.0204	**0.0052**
V1_03	**0.0043**	0.0216	0.0044	0.0074	0.0309	**0.0071**
V2_01	**0.0030**	0.0041	0.0070	**0.0049**	0.0050	0.0075
V2_02	—	**0.0059**	0.0069	—	0.0085	**0.0081**
V2_03	0.0052	0.0017	**0.0014**	0.0096	0.0082	**0.0044**

Note: bold values indicate the lowest RPE value for each metric in each row.

**Table 4:** Comparison of average front-end feature statistics on the V1_02 sequence.

Metric	VINS-Fusion	A2PM-VINS
Number of current-frame feature points	150.00	323.97
Number of continuously tracked points (≥2 frames)	103.55	232.51
Continuous tracking ratio	0.6904	0.7184
Average track length (frames)	8.22	6.43

**Table 5:** Per-sequence Absolute Trajectory Error (ATE) comparison in the Anchor–Explorer (A–E) ablation experiment.

Sequence	A2PM-VINS-CG	A2PM-VINS-AE	ATE Reduction	Reduction (%)
MH04	0.1123	0.0983	0.0140	12.47
MH05	0.1484	0.1191	0.0293	19.74
V1_02	0.1243	0.1076	0.0167	13.44
V1_03	0.1283	0.1210	0.0073	5.69
V2_02	0.1645	0.1349	0.0296	17.99

**Table 6:** Effect of A–E on feature quality, back-end optimization, and computational load.

Method	Average Retained Features	Continuous Tracking Ratio	Average Track Length	Long-Track Ratio	Ceres Iterations	Solver Time/ms	Selection Time/ms	Average ATE/m
A2PM-VINS-CG	302.5	0.759	12.13	0.255	7.26	34.05	0.29	0.1597
A2PM-VINS-AE	337.8	0.751	9.19	0.216	6.32	36.21	9.52	0.1303

**Table 7:** Absolute Trajectory Error (ATE)results under different grid sizes on each sequence.

Grid Size	MH_04	MH_05	V1_02	V1_03	V2_02	V2_03	Average
30 × 30	0.1094	0.1090	0.1245	0.2077	0.1441	0.3640	0.1765
35 × 35	0.0944	0.1161	0.1125	0.1537	0.1645	0.3318	0.1622
40 × 40	0.0983	0.1191	0.1076	0.1210	0.1349	0.2009	0.1303
45 × 45	0.0889	0.1371	0.1149	0.1643	0.1311	0.2599	0.1494
50 × 50	0.1067	0.1344	0.1202	0.1975	0.1442	0.2593	0.1604

**Table 8:** Nominal capacity, actual retained feature count, and computational cost under different grid sizes.

Grid Size	Nominal Grid-Stage Capacity	Actual Average Retained Features	Continuous Tracking Ratio	Selection Time/ms	Solver Time/ms	Ceres Iterations	Average ATE/m
30 × 30	832	500.4	0.731	9.78	37.36	5.60	0.1765
35 × 35	616	402.9	0.737	9.64	36.27	6.12	0.1622
40 × 40	456	337.8	0.751	9.52	36.21	6.32	0.1303
45 × 45	374	310.6	0.766	8.90	35.99	6.24	0.1494
50 × 50	320	299.2	0.777	8.84	36.43	5.96	0.1604

**Table 9:** Ablation results of the Sub-Window Consistency (SWC) dynamic weighting mechanism.

Sequences	A2PM-VINS	A2PM-VINS + SWC	ATE Change
MH_04	0.0983	0.0966	+0.0017
MH_05	0.1191	0.1067	+0.0124
V1_02	0.1076	0.1024	+0.0052
V1_03	0.1210	0.1481	−0.0271
V2_02	0.1349	0.1439	−0.0090
V2_03	0.2009	0.2218	−0.0209

**Table 10:** Runtime cost decomposition of each component.

Module	Statistical Unit	Main Operation	Average Time	Online/Offline	Description
Complete adjacent-frame preprocessing pipeline	Per image pair	Region segmentation, region matching, point-level matching inside regions, track inheritance, and result saving	63,073.25 ms	Offline	Complete A2PM front-end preprocessing cost in the current implementation
DMESA region matching	Per image pair	Establishing region-level correspondences	18,853.86 ms	Offline	Region-level correspondence establishment in area-to-point matching
Track inheritance mechanism	Per image pair	Tracklet association and ID inheritance	437.42 ms	Offline	Continuity association process related to historical track inheritance
CG selection	Per frame	Conventional grid-based selection	0.29 ms	Online	Internal baseline in the A–E ablation
A–E selection	Per frame	Anchor–Explorer dual-role selection	9.52 ms	Online	Feature selection module proposed in this paper
Ceres solver (without SWC)	Per optimization window	Sliding-window nonlinear solving	36.21 ms	Online	Pure solving time, excluding the complete back-end pipeline
Ceres solver (with SWC)	Per optimization window	Sliding-window nonlinear solving with SWC weighting	36.22 ms	Online	Used to measure the effect of SWC on solving cost

## Data Availability

The EuRoC MAV dataset used in this study is publicly available from its official source. The processed data generated for the A2PM-VINS pipeline, including the intermediate matching results used in the experiments, are available from the corresponding author upon reasonable request.

## References

[1] Mourikis A.I., Roumeliotis S.I. A Multi-State Constraint Kalman Filter for Vision-Aided Inertial Navigation. Proceedings of the 2007 IEEE International Conference on Robotics and Automation (ICRA).

[2] Geneva P., Eckenhoff K., Lee W., Yang Y., Huang G. OpenVINS: A Research Platform for Visual-Inertial Estimation. Proceedings of the 2020 IEEE International Conference on Robotics and Automation (ICRA).

[3] Leutenegger S., Lynen S., Bosse M., Siegwart R., Furgale P. (2015). Keyframe-Based Visual-Inertial Odometry Using Nonlinear Optimization. Int. J. Robot. Res..

[4] Qin T., Li P., Shen S. (2018). VINS-Mono: A Robust and Versatile Monocular Visual-Inertial State Estimator. IEEE Trans. Robot..

[5] Qin T., Pan J., Cao S., Shen S. (2019). A General Optimization-Based Framework for Local Odometry Estimation with Multiple Sensors. arXiv.

[6] Campos C., Elvira R., Gómez Rodríguez J.J., Montiel J.M.M., Tardós J.D. (2021). ORB-SLAM3: An Accurate Open-Source Library for Visual, Visual-Inertial and Multi-Map SLAM. IEEE Trans. Robot..

[7] Forster C., Pizzoli M., Scaramuzza D. SVO: Fast Semi-Direct Monocular Visual Odometry. Proceedings of the 2014 IEEE International Conference on Robotics and Automation (ICRA).

[8] DeTone D., Malisiewicz T., Rabinovich A. SuperPoint: Self-Supervised Interest Point Detection and Description. Proceedings of the IEEE/CVF Conference on Computer Vision and Pattern Recognition Workshops (CVPR Workshops).

[9] Sun J., Shen Z., Wang Y., Bao H., Zhou X. LoFTR: Detector-Free Local Feature Matching with Transformers. Proceedings of the IEEE/CVF Conference on Computer Vision and Pattern Recognition (CVPR).

[10] Lindenberger P., Sarlin P.-E., Pollefeys M. LightGlue: Local Feature Matching at Light Speed. Proceedings of the IEEE/CVF International Conference on Computer Vision (ICCV).

[11] Edstedt J., Athanasiadis I., Wadenbäck M., Felsberg M. DKM: Dense Kernelized Feature Matching for Geometry Estimation. Proceedings of the IEEE/CVF Conference on Computer Vision and Pattern Recognition (CVPR).

[12] Zhang Y., Zhao X. (2023). Searching from Area to Point: A Hierarchical Framework for Semantic-Geometric Combined Feature Matching. arXiv.

[13] Zhang Y., Zhao X. MESA: Matching Everything by Segmenting Anything. Proceedings of the IEEE/CVF Conference on Computer Vision and Pattern Recognition (CVPR).

[14] Wang K., Zhang C., Su D., Sun K., Zhan T. VINS-FEN: Monocular Visual-Inertial SLAM Based on Feature Extraction Network. Proceedings of the 2023 7th International Conference on Machine Vision and Information Technology (CMVIT 2023).

[15] Luo H., Liu Y., Guo C., Li Z., Song W. (2025). SuperVINS: A Real-Time Visual-Inertial SLAM Framework for Challenging Imaging Conditions. IEEE Sens. J..

[16] Sun Y., Wang Q., Yan C., Feng Y., Tan R., Shi X., Wang X. (2023). D-VINS: Dynamic Adaptive Visual-Inertial SLAM with IMU Prior and Semantic Constraints in Dynamic Scenes. Remote Sens..

[17] Fu Q., Wang J., Yu H., Ali I., Guo F., He Y., Zhang H. (2020). PL-VINS: Real-Time Monocular Visual-Inertial SLAM with Point and Line Features. arXiv.

[18] Li X., Liu C., Yan X. (2025). Robust Visual-Inertial Odometry with Learning-Based Line Features in an Illumination-Changing Environment. Sensors.

[19] Zhai S., Wang N., Wang X., Chen D., Xie W., Bao H., Zhang G. (2025). XR-VIO: High-Precision Visual Inertial Odometry with Fast Initialization for XR Applications. arXiv.

[20] Xu K., Hao Y., Yuan S., Wang C., Xie L. (2025). AirSLAM: An Efficient and Illumination-Robust Point-Line Visual SLAM System. IEEE Trans. Robot..

[21] Kirillov A., Mintun E., Ravi N., Mao H., Rolland C., Gustafson L., Xiao T., Whitehead S., Berg A.C., Lo W.-Y. Segment Anything. Proceedings of the IEEE/CVF International Conference on Computer Vision (ICCV).

[22] Zhang Y., Zhao X. (2024). DMESA: Densely Matching Everything by Segmenting Anything. arXiv.

[23] Burri M., Nikolic J., Gohl P., Schneider T., Rehder J., Omari S., Achtelik M.W., Siegwart R. (2016). The EuRoC Micro Aerial Vehicle Datasets. Int. J. Robot. Res..

[24] Grupp M. evo: Python Package for the Evaluation of Odometry and SLAM. https://github.com/MichaelGrupp/evo.

[25] Gálvez-López D., Tardós J.D. (2012). Bags of Binary Words for Fast Place Recognition in Image Sequences. IEEE Trans. Robot..

[26] Wiseman Y. Real-Time Monitoring of Traffic Congestions. Proceedings of the 2017 IEEE International Conference on Electro Information Technology (EIT).

